# Prevalence and Factors Associated with Hepatitis B Immunization and Infection among Men Who Have Sex with Men in Beijing, China

**DOI:** 10.1371/journal.pone.0048219

**Published:** 2012-10-25

**Authors:** Chao Wang, YouXin Wang, XiaoJie Huang, Xia Li, Tong Zhang, ManShu Song, LiJuan Wu, Juan Du, XiaoQin Lu, Shuang Shao, FeiFei Zhao, Michele A. Ball, Hao Wu, Wei Wang

**Affiliations:** 1 School of Public Health and Family Medicine, Capital Medical University, Beijing, China; 2 Center for Infectious Diseases, Beijing YouAn Hospital, Capital Medical University, Beijing, China; 3 Beijing Municipal Key Laboratory of Clinical Epidemiology, Beijing, China; 4 Acupuncture Department, Beijing University of Chinese Medicine, Beijing, China; 5 College of Life Sciences, Graduate University, Chinese Academy of Sciences, Beijing, China; 6 School of Medical Sciences, Edith Cowan University, Perth, Australia; University of Texas Health Science Center San Antonio Texas, United States of America

## Abstract

**Background:**

Among the Chinese population of 1.3 billion, there are an estimated 93 million carriers of hepatitis B virus (HBV). Men who have sex with men (MSM) are at increased risk for HBV infection; however, the prevalence of HBV immunization and infection among Chinese MSM remains undetermined.

**Methods:**

A cross-sectional survey of 1,114 eligible participants was conducted in Beijing, China. Multiple methods were used to recruit study participants. Demographic information and potential correlated factors were collected by questionnaire. Additionally, blood specimens were collected and tested for sexually transmitted infections and serologic markers of hepatitis B immunization and infection.

**Results:**

Laboratory data were available for 1,111 participants (99.7%), the prevalence of hepatitis B immunization was 38.9%, and 26.5% had serologic markers of HBV infection. Multivariate analyses indicated that higher education level, smaller number of male sex partners in the past 12 months, reported diagnosis of sexually transmitted disease (STD), and history of blood donation were independently associated with HBV immunization. Absence of steady male sex partner(s) in the past 12 months, and reported diagnosis of STD were shown to be independently associated with HBV infection. MSM positive for HBV infection were more likely to have past or current syphilis infection.

**Conclusions:**

Low prevalence of HBV immunization and high prevalence of HBV infection among Chinese MSM and a correlation between sexual risk factors and hepatitis B infection indicate that comprehensive preventative measures for HBV among MSM, including blood donor and HIV-STD clinic vaccination programs as well as targeted health education campaigns should be developed in China.

## Introduction

Hepatitis B virus (HBV) infection is a major global health concern due to the high morbidity and mortality associated with cirrhosis and primary liver cancer. The World Health Organization (WHO) estimates that two billion people worldwide have been infected with HBV, and more than 350 million have chronic (long-term) liver infections [1]. In the Chinese population of 1.3 billion individuals, there are estimated to be 93 million HBV carriers. Each year, 300,000 deaths are attributed to chronic hepatitis B, including deaths associated with liver cirrhosis and hepatocellular carcinoma (HCC) [2].

HBV infection is a sexually transmitted infection (STI), and is 50 to 100 times more infectious than the human immunodeficiency virus (HIV) [3]. For individuals engaging in high-risk sexual behavior, such as unprotected anal intercourse, men who have sex with men (MSM) are at an increased risk of infection with HBV, HIV, syphilis, and other STIs. To control hepatitis B, the Chinese government has implemented infant vaccination with hepatitis B vaccine as the highest priority, thereby, hepatitis B vaccine coverage (3 doses) increased from 30.0% for children born in 1992 to 93.4% for children born in 2005 [4], and hepatitis B vaccine catch-up immunization has been extended to adolescents under 15 years of age in year 2009 [5]. In order to improve the current national hepatitis B vaccine immunization strategy, evaluating hepatitis B immunization among high risk populations such as MSM is needed.

In China, previous studies on MSM have primarily focused on HIV and syphilis infection [6]–[12]. Some studies, however, have reported HBV prevalence in terms of Hepatitis B surface antigen (HBsAg) positive rates. Despite these reports, HBV infection prevalence is poorly represented by the determination of HBsAg alone [13]. Furthermore, no reports on hepatitis B immunization among Chinese MSM have been published to date.

The current study aims to: (1) assess the prevalence of HBV infection among MSM in Beijing, China; (2) explore risk factors associated with HBV infection and the association of HBV infection with other STIs (HIV, syphilis) and hepatitis C virus (HCV) infection; and (3) assess hepatitis B immunization rate and thus explore factors associated with immunization among MSM in Beijing, China.

## Materials and Methods

### Ethics Statement

This study was approved by Ethics Committee of the Beijing YouAn Hospital, Capital Medical University, Beijing, China. Written informed consent was obtained from each participant involved in this study. For participants under the age of eighteen, written informed consents were obtained from their guardians. All participants' information were kept confidential and tracked anonymously with an identification number only.

### Study Design and Population

From March 2010 to June 2011, a cross-sectional study of 1,111 eligible participants was conducted among MSM in Beijing, China. The following three strategies were used to recruit MSM into the study: (1) web-based advertisements on www.bjboy.net targeting MSM in Beijing; (2) distribution of information leaflets by trained peer recruiters at MSM-frequented venues, such as MSM bars, bathhouses, and parks; and (3) peer referral by current study participants. All potential participants participated in voluntary counseling and testing (VCT) clinic at the Beijing YouAn Hospital for eligibility assessment. Participants were screened against the following eligibility criteria: (1) aged 16 years or older; (2) self-reported anal sex with another male within the past 6 months; (3) willingness to participate in the study and to provide written informed consent; and (4) had not participated in this study previously. Potential participants meeting the above criteria were asked to participate in a face-to-face interview using a prepared questionnaire and to provide blood specimens for laboratory testing. Trained medical doctors provided all potential participants with risk reduction counseling on HIV/acquired immune deficiency syndrome (AIDS) and other sexually transmitted diseases (STDs). At this point, each eligible study participant was assigned a unique study identification code and provided with the telephone number of the research department. Thus, participants were able to anonymously retrieve blood test results within a two week period following collection. Participants testing positive for any STI (syphilis, HBV, HIV) or HCV were referred to the Beijing YouAn Hospital for treatment, and vaccination was recommended to those who were susceptible to HBV (susceptible group). Each participant was compensated 50 Chinese Yuan (equivalent to $ 7.90) for time and transportation costs.

### Data Collection

Questionnaire-based surveys were conducted by trained interviewers on a one-to-one basis. The questionnaire included the following measures on: (1) demographics such as age, education, and income; (2) sexual behaviors such as number of sexual partners, and the type of male sex partners (Steady partner(s) were defined as sole sex partners in an ongoing relationship; casual partner(s) were defined as sex partners with no social relationship); (3) potential sexual or non-sexual risk factors for STD transmission, such as history of STDs or injection drug use); and (4) history of HBV vaccination (assessed by self-report "yes/no/can not remember"). Blood specimens from study participants were collected in sterile ethylene diamine tetraacetic acid (EDTA) tubes, aliquoted, and stored at −70°C for further testing.

Each completed questionnaire was linked to its corresponding blood specimen through a unique study identification code.

### Laboratory Testing

Blood specimens were tested for the serologic markers of HBV, HIV, syphilis and HCV infections. Enzyme immunoassay screening (Kehua Bio-engineering Co., Ltd., Shanghai, China) was carried out to detect the presence of the following three serologic markers: HBsAg, antibody to HBsAg (anti-HBs), and antibody to hepatitis B core antigen (anti-HBc). Vaccine-associated immunity was defined by the sole presence of anti-HBs. Immunity due to natural infection (past infection) was defined by the presence of anti-HBc and anti-HBs with an absence of HBsAg. Susceptibility to HBV infection was defined as the complete absence of these three serologic markers. Current HBV infection, including acute infection and chronic infection, was defined by the presence of HBsAg confirmed with anti-HBc. The above interpretation of hepatitis B serologic test results was adapted from the official website of the Center for Disease Control and Prevention (CDC), USA [14] and the Committee on the Prevention and Control of Viral Hepatitis Infections, Institute of Medicine of the National Academies, USA [13]. In addition, total HBV infection was defined as the presence of HBsAg or anti-HBc.

HIV-1/2 infection status was first determined using enzyme-linked immunosorbent assay (ELISA; Diagnostic Kit for Antibody to Human Immunodeficiency Virus, Kehua Bio-engineering Co., Ltd., Shanghai, China), and positive specimens were transferred to the HIV Confirmatory Laboratory of the Beijing Center for Disease Control and Prevention. There, HIV-1/2 Western Blot tests (HIV Blot 2.2 WB™, Genelabs Diagnostics, Singapore) were used for confirmation.

The syphilis screening was performed by rapid plasma reagin (RPR; Shanghai Kehua Bio-engineering Co., Ltd., Shanghai, China) and *Treponema pallidum* particle agglutination assay conducted in parallel (TPPA; Fujirebio Inc., Tokyo, Japan). Thus, all participants were tested by TPPA, and positive TPPA cases were further tested by RPR. A seropositive result for TPPA was defined as the presence of a past or current syphilis infection, while a seropositive result for both TPPA and RPR was diagnosed as a current syphilis infection.

HCV infection status was determined by presence of antibody to HCV (anti-HCV) using ELISA (Kehua Bio-engineering Co., Ltd., Shanghai, China).

All diagnostic kits were licensed by the State Food and Drug Administration, P. R. China.

### Statistical Analysis

Questionnaire-based data and serological testing results were recorded in duplicate using the EpiData software (EpiData 3.0 for Windows, The EpiData Association, Odense Denmark). Data validation tools were also used to validate duplicate data entry.

Because of the lack of concordance between self-reported vaccination and serologic evidence of vaccination, only serologic evidence was used as the marker of vaccination for data analysis. To explore factors associated with hepatitis B vaccination, vaccine-associated immunity and hepatitis B susceptible status were compared. Those with serologic evidence of hepatitis B vaccination were unlikely to be infected with HBV, and therefore during examination of associated factors for hepatitis B infection, only data from the other groups (hepatitis B infected group and susceptible group) were included in the analysis.

For univariate analyses examining factors associated with immunization and infection, Mann-Whitney *U* tests were used for continuous data because they were not normally distributed, and χ^2^ tests were used for categorical data. The significance level was set at less than 0.05 (*P*<0.05).

To assess independent factors associated with immunization and infection, two separate multiple logistic regression analyses using forward unconditional modeling were performed. Variables included in multivariable testing were those found to be associated with the dependent variables (*P*<0.05) through univariate analyses. Both adjusted odds ratio (AOR) and 95% confidence interval (95% CI) were obtained for each explanatory variable. Variables with *P*<0.05 in multivariable analysis were considered statistically significant. Statistical analyses were performed using the Statistical Package for Social Science (SPSS) for Windows (Version 13.0; SPSS Inc., Chicago, IL, USA).

## Results

### Participants

A total of 1,260 potential participants visited the VCT clinic during the study period, they came in as a result of the three forms of recruitment and were screened for eligibility. The distribution of participants by method of recruitment was unknown. Among them, 67 refused to provide written informed consent, 79 were not eligible, and 3 did not provide suitable specimens. Analyses were restricted to 1,111 (88.2%) eligible MSM who provided specimens suitable for testing.

**Figure 1:**
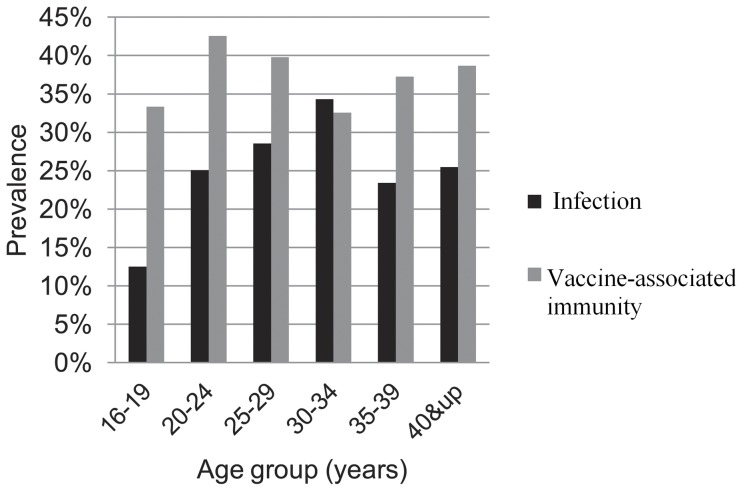
Seroprevalence of hepatitis B infection and vaccine-associated immunity among MSM by age group. For those under age 35 years, seroprevalence of hepatitis B infection increased linearly with age, reaching its lowest (12.5%) levels among participants aged 16–19 years and highest (34.3%) levels among participants aged 30–34 years (*P* = 0.001; χ^2^ test for trend). No significant difference in the vaccine-associated immunity was found among all age-groups.

Among these 1,111 participants, the prevalence of HBsAg, anti-HBc, HIV, and anti-HCV were 9.0%, 23.8%, 11.6%, and 3.9%, respectively. A total of 368 (33.1%) participants seropositive for TPPA were determined to have had past or current syphilis infections, 149 (13.4%) participants seropositive for both TPPA and RPR were diagnosed with current syphilis infections, and 34 (3.1%) participants were diagnosed with early syphilis (RPR titer ≥1∶16 with confirmatory TPPA). Table 1 presents the Hepatitis B serologic panels, and Figure 1 shows the seroprevalence of hepatitis B infection and vaccine-associated immunity by age group. For participants under age 35 years, seroprevalence of hepatitis B infection increased linearly with age, reaching lowest (12.5%) values among participants aged 16–19 years and highest (34.3%) values among participants aged 30–34 years (*P* = 0.001; χ^2^ test for trend). No significant difference in the prevalence of vaccine-associated immunity was found among variant age-groups. Selected demographic and sexual behavior characteristics of the 1,111 participants presenting valid samples are described in Table 2.

**Table 1:** Serological markers for hepatitis B infection and immunity among men who have sex with men (MSM) in Beijing, China (N = 1111).

Variable	n	%	95% CI
Total infection	294	26.5	23.9–29.1
Current infection (HBsAg+, anti-HBc+, anti-HBs-)	68	6.1	4.7–7.5
Immunity due to natural infection (HBsAg-, anti-HBc+, anti-HBs+)	164	14.8	12.7–16.9
Other infection status (e.g. HBsAg-, anti-HBc+, anti-HBs-; HBsAg+, anti-HBc-, anti-HBs+)	62	5.6	4.2–6.9
Vaccine-associated immunity (HBsAg-, anti-HBc-, anti-HBs+)	432	38.9	36.0–41.8
Susceptible (HBsAg-, anti-HBc-, anti-HBs-)	385	34.7	31.9–37.5

Abbreviation: CI, confidence interval; HBsAg, hepatitis B surface antigen; anti-HBc, antibodies to hepatitis B core antigen; anti-HBs, antibodies to hepatitis B surface antigen.

**Table 2:** Selected demographics, potential associated behaviors for hepatitis B infection, and immunization among men who have sex with men (MSM) in Beijing, China (N = 1111).

	No. (%) of Hepatitis B serologic status *			
	Total	Vaccine-associated immunity	Infection	Susceptible
Characteristics	N = 1111	n = 432	n = 294	n = 385
Median age (range)	26 (16–66)	25 (16–57)	27 (17–56)	26 (16–66)
Age categories				
16–19	72	24 (33.3)	9 (12.5)	39 (54.2)
20–24	383	163 (42.6)	96 (25.1)	124 (32.4)
25–29	284	113 (39.8)	81 (28.5)	90 (31.7)
30–34	172	56 (32.6)	59 (34.3)	57 (33.1)
35–39	94	35 (37.2)	22 (23.4)	37 (39.4)
>39	106	41 (38.7)	27 (25.5)	38 (35.8)
Education level				
Junior high school or less	272	80 (29.4)	84 (30.9)	108 (39.7)
Senior high school	398	149 (37.4)	106 (26.6)	143 (35.9)
Some college	318	136 (42.8)	77 (24.2)	105 (33.0)
Graduate college	123	67 (54.5)	27 (22.0)	29 (23.6)
Married/cohabited				
No	873	356 (40.8)	228 (26.1)	289 (33.1)
Yes	236	75 (31.8)	66 (28.0)	95 (40.3)
Student status				
No	1029	387 (37.6)	279 (27.1)	363 (35.3)
Yes	82	45 (54.9)	15 (18.3)	22 (26.8)
Number of male sex partners in the past 12 months				
≤3	700	291 (41.6)	180 (25.7)	229 (32.7)
>3	411	141 (34.3)	114 (27.7)	156 (38.0)
Presence of steady male sex partner(s) in the past 12 months				
No	966	378 (39.1)	268 (27.7)	320 (33.1)
Yes	145	54 (37.2)	26 (17.9)	65 (44.8)
History of injected drug use				
No	1106	431 (39.0)	290 (26.2)	385 (34.8)
Yes	5	1 (20.0)	4 (80.0)	0 (0)
Sexually transmitted disease, ever				
No	1016	392 (38.6)	257 (25.3)	367 (36.1)
Yes	95	40 (42.1)	37 (38.9)	18 (18.9)
History of blood donation				
No	884	325 (36.8)	241 (27.3)	318 (36.0)
Yes	227	107 (47.1)	53 (23.3)	67 (29.5)

* Totals reflect valid data submissions and may not always equal the total as a result of missing data in individual categories.

### Magnitude and Correlates of Vaccine-associated Immunity

A total of 432 (38.9%) participants had serologic evidence of hepatitis B vaccination (vaccine-associated immunity), 385 (34.7%) participants were susceptible to HBV infection (Table 1), and 164 (14.8%) participants recalled a history of HBV vaccination. Both HBV vaccination history and serologic evidence of vaccination were observed in 80 (7.2%) participants. Vaccine-associated immunity was observed in 80 (48.8%) who recalled a history of HBV vaccination. Overall, 766 (68.9%) participants expressed uncertainty about their history of HBV vaccination.

In the univariate analyses, prevalence of vaccine-associated immunity increased with increasing education level, being the lowest (42.6%) levels among those with junior high school diplomas or lower and the highest (69.8%) levels among those with graduate college diplomas (*P*<0.001; χ^2^ test for trend). Furthermore, men with serologic evidence of hepatitis B vaccination were more likely to be single, possess student status, have had no more than 3 male sex partners in the past 12 months, have reported STD diagnosis, and have a history of donating blood. In a binary multivariate analysis using the forward logistic regression model, four factors remained significantly associated with hepatitis B vaccine-associated immunity: higher education level (AOR = 1.60, 95% CI: 1.08–2.38, some college vs. junior high school or less; AOR = 2.88, 95% CI: 1.69–4.91, graduate college vs. junior high school or less); more than 3 male sex partners in the past 12 months (AOR = 0.73, 95% CI: 0.54–0.98); reported STD diagnosis (AOR = 2.21, 95% CI: 1.23–3.98), and history of blood donation (AOR = 1.42, 95% CI: 1.00–2.03) (Table 3).

**Table 3:** Factors associated with vaccine-associated immunity among non-HBV infected men who have sex with men (MSM) in Beijing, China (N = 817).

Variables	No. vaccine-associated immunity/tested (%)	OR (95% CI)	AOR (95% CI)
Married/cohabiting with sex partners †			
No	356/645 (55.2)	1.00	
Yes	75/170 (44.1)	0.64 (0.46–0.90)§	NS
Education level			
Junior high school or less	80/188 (42.6)		1.00
Senior high school	149/292 (51.0)		1.40 (0.96–2.04)NS
Some college	136/241 (56.4)		1.60 (1.08–2.38)§
Graduate college	67/96 (69.8)	¶ ‡	2.88 (1.69–4.91)‡
Student status			
No	387/750 (51.6)	1.00	
Yes	45/67 (67.2)	1.92 (1.13–3.26)§	NS
Number of male sex partners in the past 12 months			
≤3	291/520 (56.0)	1.00	1.00
>3	141/297 (47.5)	0.71 (0.53–0.95)§	0.73 (0.54–0.98)§
Sexually transmitted disease, ever			
No	392/759 (51.6)	1.00	1.00
Yes	40/58 (69.0)	2.08 (1.17–3.69)§	2.21 (1.23–3.98)*
History of blood donation			
No	325/643 (50.5)	1.00	1.00
Yes	107/174 (61.5)	1.56 (1.11–2.20)§	1.42 (1.00–2.03)§

Abbreviation: OR, odds ratio; CI, confidence interval; AOR, adjusted odds ratio.

* *P*<0.01; ‡ *P*<0.001; § *P*<0.05; NS indicates no statistical significance.

¶ χ^2^ test for trend.

† Totals reflect valid data submissions and may not always equal the total as a result of missing data in individual categories.

Univariate analyses indicate that prevalence of HBV immunization did not vary by age, ethnicity, native place, personal income, having steady male sex partner(s) in the past 12 months, or history of injected drug use.

### Magnitude and Correlates of HBV Infection

Among the 1,111 participants, a total of 294 (26.5%) had serologic evidence of HBV infection, 68 (6.1%) were currently HBV infected, and 164 (14.8%) were immune due to natural infection (Table 1).

Univariate analysis identified 3 factors associated with hepatitis B infection, including absence of steady male sex partners in the past 12 months, reported STD diagnosis, and history of injected drug use. Multivariate analysis using forward stepwise logistic regression confirmed that the presence of steady male sex partner(s) over the past 12 months (AOR = 0.50, 95% CI: 0.31–0.82) and reported STD diagnosis (AOR = 2.79, 95% CI: 1.55–5.03) were factors associated with hepatitis B infection (Table 4).

**Table 4:** Factors associated with hepatitis B infection among non-immunized men who have sex with men (MSM) in Beijing, China (N = 679).

Variables	No. infection/tested (%)	OR (95% CI)	AOR (95% CI)
Presence of steady male sex partner(s) in the past 12 months			
No	268/588 (45.6)	1.00	1.00
Yes	26/91 (28.6)	0.48 (0.30–0.77)*	0.50 (0.31–0.82)*
Sexually transmitted disease, ever			
No	257/624 (41.2)	1.00	1.00
Yes	37/55 (67.3)	2.94 (1.64–5.27)‡	2.79 (1.55–5.03)*
History of injected drug use			
No	290/675 (43.0)	1.00	
Yes	4/4 (100.0)	¶ §	NC

Abbreviation: OR, odds ratio; CI, confidence interval; AOR, adjusted odds ratio; NC: not computed by SPSS.

* *P*<0.01; ‡ *P*<0.001; § *P*<0.05.

¶ Fisher's exact test.

In addition, participants with serologic evidence of HBV infection were more likely to have had past or current syphilis infections (OR = 1.48, 95% CI: 1.07–2.04, χ^2^ test). Univariate analyses indicate that prevalence of HBV infection did not vary by the serologic test outcomes of HIV and HCV, ethnicity, education level, native place, personal income, marital status, student status, number of male sex partners in the past 12 months, or history of blood donation.

## Discussion

A novel examination of the prevalence and factors associated with hepatitis B immunization and infection among a convenience sample of the MSM population in Beijing, China was conducted. The findings from this study suggest that a large portion of the Chinese MSM population are still not vaccinated against hepatitis B, despite the increased availability and affordability of the vaccine. In fact, as little as 38.9% of MSM participants examined in the current study presented evidence of prior vaccination, additional work is required to raise awareness and increase participation in vaccination programs.

In 1992, the hepatitis B vaccination was first introduced in China, where its use was progressively expanded during the following years. After nearly two decades of effort, the HBsAg positive rate in the general population of China dropped from 9.75% in 1992 to 7.18% in 2006 [4]. In response to this relatively slow reduction in infection rates, a hepatitis B vaccine catch-up immunization program was initiated in 2009 and extended to all children and adolescents in China under 15 years of age [5]. Therefore, it is likely that the current prevalence of HBsAg in the general population may have decreased as vaccination coverage in both children and adolescents increased. Notably, the prevalence of HBsAg among MSM participants in the current study was 9.0%, a level notably higher than that observed in the general population.

Based on an epidemiological study among the general population in Beijing, the HBsAg prevalence was found to be 3.8% (176/4625) among participants over 15 years of age in year 2006 [15]. The HBsAg prevalence in the current study (9.0%) was much higher than that of general population in Beijing. While maternal-infant transmission of HBV infection was estimated to account for 40–50% of HBV carriers in Chinese populations [16], no evidence exists to suggest that there are different maternal-infant transmission patterns of HBV infection between the general population and MSM. Therefore, other modes of HBV transmission are likely to be involved in the increased HBsAg prevalence among this MSM population. Consistent with previous research [17]–[22], previous injection drug use and reported diagnosis of STD were associated with increased HBV infection. In addition, the status of having no steady male sex partner(s) in the past 12 months were shown to be independently associated with HBV infection. Furthermore, men with HBV infection were more likely to have past or current syphilis infections, further corroborating that sexual behavior is an important mode of HBV transmission among MSM. Although further research is required to assess the modes of transmission affecting MSM populations and the differences between the prevalence of such transmission modes between MSM and general population, these findings suggest that mode of transmission may be important in design of vaccination and education programs.

The HBsAg positive rate in the current study was similar to, or higher than, the HBsAg positive rates previously reported in other studies targeting MSM populations in Beijing, China (7.5% in 2004, 8.0% and 8.8% in 2005, 10.3% in 2006, and 6.5% in 2007) [7], [8], [23], demonstrating the ongoing transmission of HBV. Nearly one of every three MSM in the current study were susceptible to HBV infection, and no HBV vaccination programs specifically targeting MSM in Beijing currently exist, which may be a primary causative factor in the ongoing transmission of HBV among MSM [24], [25].

Several studies have reported hepatitis B immunization rates among MSM in the United States (USA) (9% in 7 US metropolitan areas between 1994 and 1998 [26], 24.5% in King County, Washington between 1997 and 2000 [18], and 17.2% in 6 other US metropolitan areas between 1998 and 2000 [27]). Although the hepatitis B immunization rate reported here (38.9%) was higher than those reported in the USA, it cannot be assumed that prevalence of hepatitis B immunization among MSM populations in China is higher than that in the USA, particularly in consideration of the fact that the majority of USA studies available were carried out many years earlier than the current study. Thus, these studies, while informative, are unlikely to reflect the contemporary success of these growing vaccination programs. A finding of higher hepatitis B immunization rates among MSM in China with higher educational levels, consistent with similar studies conducted in the USA [26], was encouraging. These studies indicated that vigorous health education campaigns may be a critical component in the successful promotion of vaccination in MSM populations in China. Furthermore, blood donation was independently associated with immunization, indicating that some MSM knew their HBV susceptible status, likely from testing by the blood bank. Such indicators likely result in increases in voluntary vaccination due to increased health awareness, although other explanations for this behavior may also exist. Reporting a diagnosed STD was a predictor associated with both HBV infection and HBV immunization, suggesting that some MSM subjects acquired HBV infection through high-risk sexual contact, whereas others sought vaccination due to previous infection with other STDs. Thus, vaccination requirements for blood donors and vaccination programs in HIV-STD clinics may also be effective alternative methods for improving immunization rates in the general population and, specifically, in MSM populations.

Of particular concern, participants who had more than 3 male sex partners in the past 12 months showed lower immunization rates (47.5%) compared with those who had between 1 and 3 partners (56.0%) during the same time period. More male sex partners generally represents more opportunities for infection. The current findings indicate that the seroprevalence of hepatitis B infection increased linearly in sexually active age-groups (Figure 1), underscoring the increasing need for health programs to aid in the early identification and vaccination of at risk populations, such as MSM. It is difficult, however, to identify such a target group due to the failure of many MSM to report their sexual orientation to health providers. Strategies for the achievement of a wider HBV vaccination coverage among Chinese MSM should be a priority for public health. Because many MSM individuals do not realize the risks or severe clinical consequences associated with STIs, raising awareness and increasing risk perception should be incorporated into vaccination programs in order to effectively limit the spread of viral hepatitis among MSM populations [28].

Interestingly, the conspicuous drop in prevalence of HBV infection among MSM above 34 years of age is shown in Figure 1. MSM above 34 years of age were less sexually active than those under 34 years of age and therefore were at lower risks for HBV infection through high-risk sex activities, which might account for this finding (data not reported).

The results of the present study should be interpreted with caution due to the limitations involved in the relatively small sample size and representativeness of the sample investigated. The recruitment methods produced a convenience sample of MSM whose representation of any broader population of MSM in Beijing is unknown; nonparticipants may possess different demographic profiles or risk behaviors, indicative of variant STI prevalence. Thus, within the present study may have resulted in some degree of overestimation or underestimation of the true prevalence of hepatitis B infection and other STIs in the Chinese MSM population. Furthermore, questionnaire data relies heavily on accurate retrospective self-reporting, a method inevitably subject to some error due to both recall bias and the sensitive nature of questions, such as those pertaining to drug use and sexual behavior. Participant concern with the social desirability of certain answers may be responsible for some error, though care was taken to ensure anonymous data collection and thus encourage accurate questionnaire responses. Also, due to the use of only serologic evidence as the marker for vaccination, the report of vaccine-associated immunity may have been misclassified. Vaccine-induced anti-HBs may have waned below detectable levels among some MSM who recalled a history of HBV vaccination, which may explain the variation observed between self-report data and serologic evidence of vaccination. Furthermore, for some men without a vaccination history, very low exposure to HBV may have resulted in the development of anti-HBs without infection [29].

In conclusion, low prevalence of vaccine-associated immunity and high prevalence of HBV infection were observed among the MSM population in Beijing, China. This study suggests that high-risk sexual contact is an important mode of HBV transmission among MSM individuals. It must be noted, however, that sexual behavior characteristics correlated with hepatitis B infection do not necessarily represent an association. This is due to the fact that hepatitis B may have been acquired before the period of assessment, and thus only a chance relationship exists. Hepatitis B vaccination for all infants has been provided for free since 2005 in China [4], but no official policy has been formulated to promote hepatitis B vaccination among MSM and other high-risk sexually active populations. Despite the ready availability of hepatitis B vaccination at a relatively low price (currently, approximately 20 Chinese Yuan, equivalent to $ 3.16 per dose, domestic), these results show that a considerable portion of the MSM population in Beijing, China were not vaccinated, thus remaining at risk for HBV infection. Comprehensive preventative measures for HBV among MSM should be developed. As primary strategies, hepatitis B vaccination requirements for blood donors and vaccination programs in HIV-STD clinics are recommended. In addition, targeted promotion of health education campaigns among MSM populations may raise awareness and thus increase voluntary vaccinations.
